# Multiscale Interface Engineering for Orthopedic and Dental Implants: A Review

**DOI:** 10.3390/jfb17040178

**Published:** 2026-04-03

**Authors:** Fiza Ashraf, Ataf Ali Altaf

**Affiliations:** 1 Department of Chemistry, University of Sahiwal, Sahiwal 57000, Pakistan; fizaashraf9@icloud.com; 2 Institute of Chemistry, Khwaja Fareed University of Engineering and Information Technology, Rahim Yar Khan 64200, Pakistan

**Keywords:** multiscale interface engineering, orthopedic and dental implant surfaces, nano-textured bioactive coatings, antimicrobial and immune-modulatory implants, long-term clinical performance of implants

## Abstract

Multiscale interface engineering has influenced the engineering of orthopedic and dental implants through the integration of macroscale architecture, micro-textured surfaces and nanoscale bio-cues. These characteristics help to increase mechanical stability and support early biological responses, as well as increase resistance to microbial colonization. Multiscale interface engineering also helps to explore fabrication schemes that facilitate load-sharing lattices and micro-roughened attachment zones, as well as immune-interactive nano-chemistry. In this study, the biological responses of protein adsorption, osteogenic differentiation, connective-tissue sealing, and macrophage polarization are investigated, together with functional barriers in stress transfer, fatigue resistance and biofilm control. New clinical data with regard to arthroplasty and dental implantology are reviewed to put these factors into perspective. Even though engineered surfaces are reliable in promoting early fixation and initial osseointegration, in the long term, their performance depends on the host’s biological variability, the mechanical forces of loading, coating integrity and peri-implant microbial pressure. Altogether, multiscale interface engineering is an evolving approach to enhancing the lifespan of implants and facilitating biologically sound skeletal and oral reconstruction. A structured literature search was conducted using PubMed, Web of Science, Scopus, and Google Scholar to identify studies published between 2000 and 2025. Approximately 320 articles were initially identified, of which about 140 relevant publications were selected for detailed review.

## 1. Introduction

The biological and mechanical stability of the implant–tissue interface is the key factor in the long-term success of both orthopedic and dental implantology [1]. Whether by being installed in the load-bearing bone or being exposed to the microbial challenges of the oral cavity, implants have to orchestrate cellular adhesion, bone formation, soft-tissue sealing, immune equilibrium, and biofilm colonization resistance [2]. The failure modes that occur in cases where these processes are not in harmony are micromotion, inflammatory bone loss, and peri-implant tissue breakdown [3]. This natural vulnerability has shifted recent studies that characterize this as a simple material choice into ones that need to design interfaces that interact with biology on the macro-, micro-, and nanoscales.

### 1.1. Background and Clinical Need

Orthopedic implants have been instrumental in the treatment of degenerative joint disease, fractured bones, spinal diseases, and skeletal deformities, ensuring restoration of motion, loading and structural alignment [4]. Their functional lifespan is related to their long-term anchorage in bone, which influences metabolism and can be continuously remodeled under the influence of mechanical stress [5]. Dental implants also cause similar rigid fixation in alveolar bone, mastication and facial stability, but they are forced to work in a highly vulnerable natural habitat for bacteria [6].

Clinical pressure remains high, with implants being regularly inserted in patients with systemic inflammation, osteoporosis, metabolic disease, vascular compromise due to smoking, or oral dysbiosis [2,7]. Recent clinical evidence indicates that implant survival in medically compromised patients requires careful risk assessment. In particular, patients receiving antiresorptive therapy for osteoporosis may have an increased risk of medication-related osteonecrosis of the jaw (MRONJ) and impaired healing outcomes [8]. These conditions slow down wound maturation, diminish mineral deposition, worsen immune signaling, and make the jaw prone to infection. There is growing evidence among younger and more active people that implants need to be able to endure decades of cyclic loading and biologic remodeling [5]. The general clinical imperative, thus, is not only biocompatibility with surfaces with the ability to stimulate quicker bone development but also stabilizing the soft tissue and suppressing deviations in inflammatory reactions and the risk of revision surgeries [9,10].

### 1.2. Limitations of Current Orthopedic and Dental Implant Interfaces

Traditional implant surfaces are mostly created based on machining accuracy, resistance to corrosion, and inert tolerance, and not on biological guidance [11]. Smooth or low-topographic titanium surfaces promote contact osteogenesis with no in-grown bone topographical stimuli [12]. The resultant effect is a mechanically constrained interface that is prone to shear displacement, micromotion and stress amplification [13]. These insufficiencies are associated with aseptic loosening and periprosthetic osteolysis in hip and knee reconstruction, induced by unstable fixation and the sustained activation of osteoclastic-mediated resorption.

In comparison, the dental implant interface problem is an analogous and demanding one. Dental implants must possess an irregular endosteal surface that promotes predictable osseointegration, while the transmucosal collar should help establish an antimicrobial barrier that limits microbial colonization [14,15]. Before implantation, plaque cannot expand since polished collar areas limit proliferation, whereas, when the connection between soft tissue is inhibited, epithelial margins are exposed, and bacteria are free to enter, as discussed in [16]. When microorganisms are organized into biofilms on this interface, the resulting inflammatory response that grows into peri-implantitis undermines bone anchorage and implant functions [17]. In orthopedics and dentistry, a cascade of biological breakdowns is permitted to occur because there are no hierarchical cues in either condition, which both include random protein adsorption, disordered integrin signals, unresolved macrophage activation, and fibro-inflammatory encapsulation [18,19].

Conventional interfaces act as passive surfaces waiting to receive acceptance by the host tissue [20]. They are not involved in controlling lineage specification, controlling immune polarity or preventing microbial establishment [21,22]. Consequently, their biological variability turns out to be one of the primary contributors to clinical outcomes instead of the foreseeability of engineered outcomes.

### 1.3. Emergence of Multiscale Interface Engineering

Multiscale interface engineering has been developed to substitute passive biological acceptance with active biological guidance [23]. Pore networks, graded stiffness constructs, and lattice geometries at the macro level can be used to promote mechanical interlocking and redistribute compressive stress in a more bone-anisotropic manner [24,25]. The roughness and topographical patterning of the microscale surface result in osteoblast adhesion, enhancement of extracellular matrix organization, and improvement of the strength of interfacial shear [26]. Rough surfaces enhance stable fibrin scaffolds, fast-track osteoid deposition, and help mineralized bone maturation [27]. Microarchitectural signals also affect the orientation of fibroblasts and anchorage of collagen fibers, which is also important for mucosal sealing of dental systems [28].

Nanoscale artificial structures have biochemical properties. The presence of nanoscale ridges, pits, and grain structures affects protein conformation, integrin clustering, cytoskeletal tension, and subsequent gene expression that governs osteogenesis and angiogenesis [29]. Nanoscale chemistry is also beginning to find applications in controlling the immune phenotype to induce macrophage conversion to pro-healing M2, as opposed to inflammatory M1 [30,31]. Modern interfaces are also intended to incorporate ion-releasing coatings, antimicrobial materials, biofilm-resistant chemistries, and peptide motifs [32]. These extensions make the implant an active signaling substrate, capable of resisting microbes, stabilizing soft tissues, and being immune-compatible.

**Figure 1:**
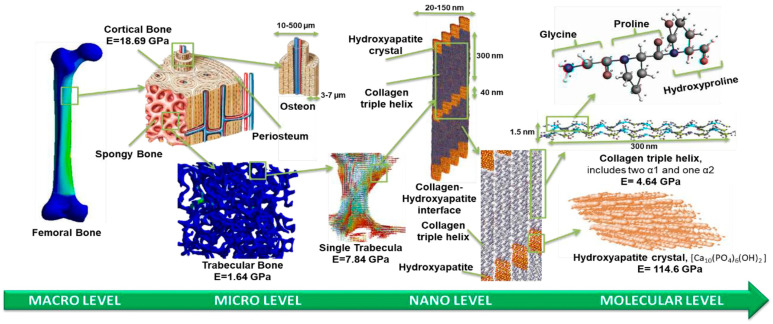
Hierarchical bone micro- to nano- and molecular-level structures, showing the multiscale mechanical and biochemical environment to which interface engineering of implants is intended to be comparable. Reprinted from Ref. [35]. CC BY 4.0 © 2024 MDPI, Basel, Switzerland.

The interplay between macro-mechanics, micro-architecture, and nano-signaling has changed the orthopedic and dental philosophy: implants are now moving beyond a fixed but inert device towards biological regeneration enhancers [33,34]. Figure 1 shows the hierarchical structure of bone at the macro-, micro-, and nanoscale levels.

### 1.4. Objectives and Scope of the Review

This review synthesizes the scientific rationale and technological, translational, and clinical implications of multiscale interface engineering to enhance osseointegration, soft-tissue stability, mechanical endurance, and biologic failure resistance in orthopedic and dental applications. This highlights the fact that the incorporation of macro-mechanical architecture, micro-textured attachment regions, and nano-biochemical signaling is repositioning implant surfaces from passive contact materials to bioactive interfaces that incorporate more deeply into host environments in terms of healing and long-term host integration.

### 1.5. Literature Search Strategy

A structured literature search was conducted to identify relevant publications on multiscale interface engineering for orthopedic and dental implant applications. Electronic databases, including PubMed, Web of Science, Scopus, and Google Scholar, were systematically searched for articles published between 2000 and 2025.

The search used combinations of keywords such as “implant surface engineering,” “multiscale interface,” “orthopedic implants,” “dental implants,” “nano-textured surfaces,” “osseointegration,” “bioactive coatings,” and “antimicrobial implant surfaces.” The reference lists of relevant review articles and research papers were manually screened to identify additional studies.

Priority was given to peer-reviewed articles focusing on biological mechanisms, surface modification technologies, fabrication strategies, clinical outcomes, and translational perspectives of implant-interface engineering. Studies not directly related to implant surface design or lacking sufficient methodological details were excluded.

This approach enabled a comprehensive synthesis of the current advances in multiscale interface engineering in orthopedic and dental implant applications.

The literature screening and selection processes followed commonly adopted systematic review practices to ensure transparency in article inclusion and exclusion.

## 2. Biological Foundations of Multiscale Implant Interfaces

The conceptual framework of multiscale interface engineering is hierarchical biological systems, where the tissue structure interfaces with biochemical signaling and mechanical behavior at organ-, tissue-, cellular-, and molecular-length scales [36,37]. To replicate these gradients in artificial implants, one must learn how natural interfaces can tolerate force transfer, cellular organization, and exposure to microbes.

### 2.1. Hierarchical Interfaces in Bone and Oral Tissues

**Figure 2:**
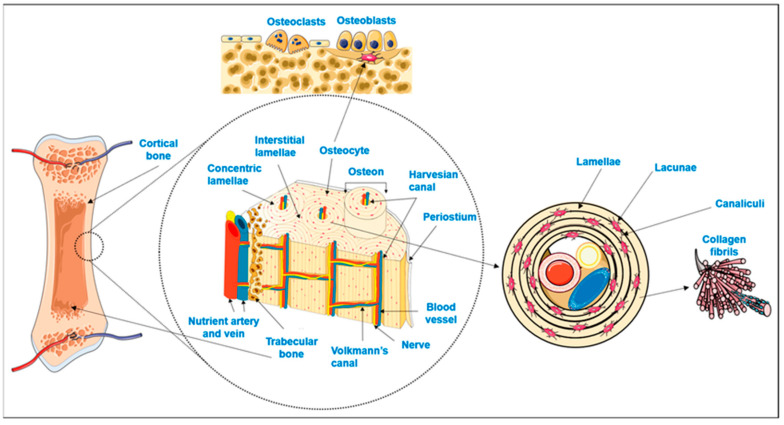
The hierarchical structure of bone with a cortical–trabecular structure, osteonal micro-architecture, and collagen–fibril nanoscale assemblies collectively regulate load distribution, bone modeling and cellular signaling in the multiscale interface design of implants. Reprinted from Ref. [42]. CC BY 4.0 © 2024 MDPI, Basel, Switzerland.

The structural hierarchy within the bone forms a framework that can offer concurrent rigidity, toughness, and remodeling capacity [36]. Mechanical signals regulate osteoblast and osteoclast activity and are transduced by the anisotropic porosity of the cortical and trabecular bones through compressive and tensile stress [38]. Lamellar structures, osteonal structures, and vascular fissures form a dynamic structure at the microscale during remodeling and mineral exchange [39]. The biochemical and mechanical contexts of integrin binding, matrix deposition, and mineral nucleation are provided by assemblies of collagen fibrils and mineral crystals on the nanoscale [40,41]. These hierarchical levels operate interdependently; the biological and mechanical changes of a particular scale occur at another scale. Figure 2 shows the multilevel interaction between the mechanical signal and structural hierarchy.

The oral peri-implant soft tissue delivers a stratified lining that controls the accessibility of bacteria and wound remodeling [43]. The epithelial seal is sensitive and prevents microbial invasion, and the connective tissue in the subepithelium is arranged in the form of collagen fibers, which aid in stabilizing the abutment and executing functional loading [44]. The integrity of these barriers relies on cell adhesion, immune surveillance, and the arrangement of the extracellular matrix [45]. In the absence of topographical or biochemical cues, fibroblasts orient parallel to, as opposed to perpendicular to, the surface, leading to less soft-tissue adhesiveness and bacterial tissue ingress through the sulcus [46,47]. The integrated processes between bone, connective tissue, epithelium, and immune regulators underscore the fact that implants should not be based on passive tolerance but on integrated multiscale biological signal delivery [48].

### 2.2. Principles of Macro–Micro–Nano Interface Design

Multiscale interfacial design aims to control biological responses by introducing structural and biochemical signals at dimensions perceived by cells [49]. Porosity, channel orientation, and graded stiffness at the macro level are used to distribute the load, decrease stress concentrations, and leave space in the volumetric space, allowing for vascular ingrowth [50]. Macro-architectures can transform surface-dependent implants into three-dimensional regenerative scaffolds to minimize micromotion and enhance anchorage under cyclic loading [51,52].

Microscale roughness activates osteoblasts, fibroblasts, and endothelial cells in response to variations in surface energy, focal adhesion, and initial matrix assembly [27]. Microfeatures enhance fibrin retention, osteoid maturation, and collagen deposition, enhancing interfacial resistance against shear forces [53]. Micro-textured collars in dental applications facilitate the maintenance of fibroblast stability and promote the formation of a connective tissue barrier with the necessary ability to resist sulcular invasion [54].

Nanoscale variations have a molecular-level effect on protein adsorption, integrin clustering, cytoskeletal organization, and expression of downstream genes [29]. Nanoscale topographies can induce the shifting of macrophages to M2 pro-regenerative states, enhance alkaline phosphatase in osteoblasts, stimulate angiogenic signaling, and increase the rate of mineral nucleation [55,56]. With the coordination of macro-, micro-, and nano-elements, the interface starts to act similarly to biological tissue, that is, it is mechanically adaptive, immunologically stable, and structurally regenerating [57].

### 2.3. Biological Responses at Engineered Interfaces

Host proteins create a conditioning layer immediately after implantation and control further cellular interactions [58]. Protein conformation is affected by surface energy, charge, and nanoscale geometry, which dictate whether adsorbed proteins display an integrin-binding motif or unfold into proinflammatory ligands [59,60]. These protein pre-adsorbed patterns preferentially favored osteogenic over fibrotic signaling before cell adhesion to the surface.

These cues are perceived by cells both mechanically and biochemically. Osteoblasts require adhesion sites that instigate intracellular tension and osteogenic signaling [61]. Fibroblasts require clues that can direct collagen fiber positioning, whereas endothelial cells require clues that enable matrix invasion and remodeling and the development of microvascular networks [62]. Macrophage polarization is one of the primary processes of immune regulation, in which M1 polarization promotes inflammatory fibrosis and osteolysis, and M2 polarization promotes regeneration and angiogenesis to maintain tissue homeostasis [63]. Nanoscale patterning, ion release, and chemical motifs have been reported to control immune decisions [64]. Dental immune–epithelial communication at the transmucosal collar plays a role in either resisting or succumbing to microbial assault on the soft tissue barrier.

Finally, engineered interface biological responses are time-dependent and hierarchical in nature. Protein adsorption regulates cell adhesion, which in turn regulates tissue organization, promoting mechanical stability and immune homeostasis [65]. Multiscale design biases these processes toward regeneration, as opposed to chronic inflammation or bacterial colonization [66]. Table 1 provides a summary of the principles of hierarchical design and important functional results at each scale.

## 3. Evolution of Implant Surface Engineering

Multiscale interface engineering did not emerge suddenly; it was the result of decades of gradual development in machining, grit-blasting, acid-etching, biochemical coating, and nanoscale manufacturing [69]. The first surfaces of the implants were designed to achieve mechanical stability and corrosion resistance. This was indicative of a time when osseointegration was passive [70]. As clinical experience with orthopedics and dentistry increased, implant surfaces were no longer considered inert metals but active biological initiators [71]. Modern implant-surface design is based on the shift from smooth machined surfaces to micro-rough surfaces, and then to biomimetic and nano-engineered surfaces.

### 3.1. Early Orthopedic and Dental Implant Surfaces

The first orthopedic devices were designed to focus on geometrical fit, precision of machining, and mechanical friction for fixing [72]. Bone formation was limited to a thin layer of apposition, and mechanical interlock was avoided by surface smoothness to form interfaces that were likely to shear off or become loosened over a long period [73]. Smooth metal interfaces provide structural stability but do not provide any biological engagement in fracture fixation hardware and spinal constructs, providing all healing responses to host variability [74].

The same was true for dental implants. Early titanium dental implants retained machined surfaces, which were supposed to minimize contamination during production, but these provided very little roughness where osteoblasts could attach [75]. Bone bonding is tedious, contact between the bone and implant is minimal, and early loading often leads to fibrous encapsulation [76]. The success of implants is highly dependent on patient biology, surgeon qualification and prolonged healing [77]. The initial era was characterized by the view that titanium had to be accepted by the host rather than actively guiding the healing process.

### 3.2. Bioactive and Micro-Topographic Surface Era

Critical changes occurred after mechanical interlocking and osteogenic stimulation, which were correlated with controlled micro-roughness [78]. Blasting and acid-etching were added to produce reproducible micro-topographies, which improved fibrin retention, osteoblast adhesion, and extracellular matrix development [79]. Porous coatings and plasma-sprayed materials produce uneven and rough surfaces in orthopedics, which enhance fixation by promoting bone growth as opposed to direct contact [80]. Such changes made the implant an active wedge rather than a passive one, enabling the textured anchor to withstand shear forces and enhance the formation of minerals.

**Figure 3:**
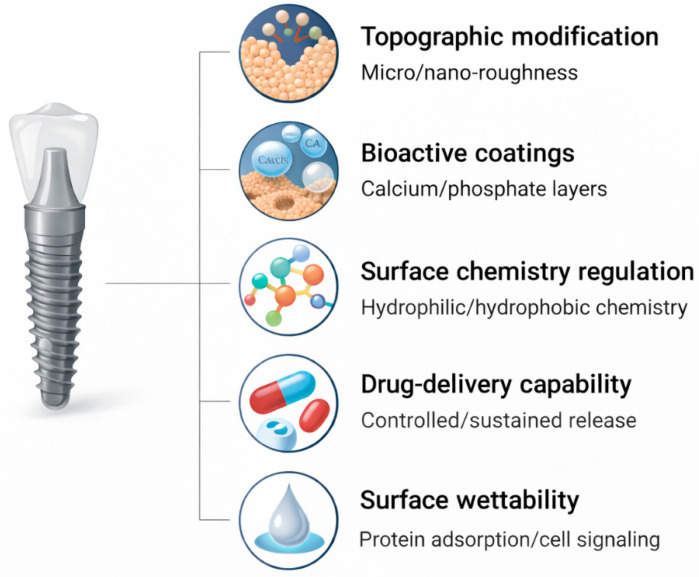
Conceptual schematic showing major surface-engineering strategies for improving the biological integration of orthopedic and dental implants, including micro/nano-roughness, bioactive coatings, surface chemistry modification, drug delivery capability, and surface wettability (figure created by the authors).

Dentistry facilitated this change with the conceptualization of micro-rough surfaces as a condition for predictable micro-osseointegration [81]. Standardized sandblasting and etched surfaces enhance bone-to-implant contact and allow for earlier loading of prostheses [82]. The healing time was also reduced because the interface could independently stabilize biologically and not mechanically [83]. With the adoption of micro-topography, the discipline had realized that the implant surface would be in a position to influence cell adhesion, cytokine production, and bone maturation, as opposed to merely sealing structural defects [84,85]. These surface-engineering strategies are conceptually summarized in Figure 3, which illustrates the representative approaches used to modify implant surfaces across multiple length scales.

Bioactive chemistries, such as calcium-phosphate-based coatings aimed at replicating the mineral phases of bone, were also introduced during this period [86]. They differed in long-term mechanical robustness across platforms, although this created a new conceptual change: chemical cues could be used in addition to geometric ones. Surface properties can be adjusted, as opposed to being fixed [87,88].

### 3.3. Nano-Engineered and Biomimetic Interfaces

Nanoscale design evolved because it was understood that cells do not communicate with micro-architecture; they detect features that are near the scale of proteins, adhesion ligands, and mineral clusters [64,89]. Nanopatterning presents a pathway to control integrin binding, cytoskeletal arrangement, and gene expression [90]. Nano-structured titanium oxide, layers of ion-modifying, and molecular coating assistance have been used in orthopedics to regulate immune polarization and osteogenic differentiation [91,92]. These functions were aimed at pathways for bone reconstruction and inflammatory control, dealing with the mechanisms of failure linked with particle-mediated osteolysis and chronic host responses.

Nanoscale methods in dental contexts have been applied to soft-tissue interface engineering [71]. Solutions to the challenges not found in orthopedic stems include engineered collars with fibroblast attachment, antibacterial chemistry, and epithelial stability [93]. The design space continues to expand through biomimetic design methods inspired by structural messages based on the bone extracellular matrix and peptide sequences of adhesion or antimicrobial barriers [94,95]. The interface was turned into a programmable environment that could control biological interactions and not just survive there.

The development of smooth surfaces to micro-rough, bioactive, and nanoscale surfaces is a gradual evolution of the biological approach [71]. The functional intention in each period was new: mechanical tolerance into mechanical anchorage, mechanical anchorage into biological stimulation, and biological stimulation into hierarchical control [96]. These changes provide an environment for completely integrated multiscale engineering. A comparative overview of these technological developments is presented in Table 2.

## 4. Fabrication Strategies and Material Platforms

**Figure 4:**
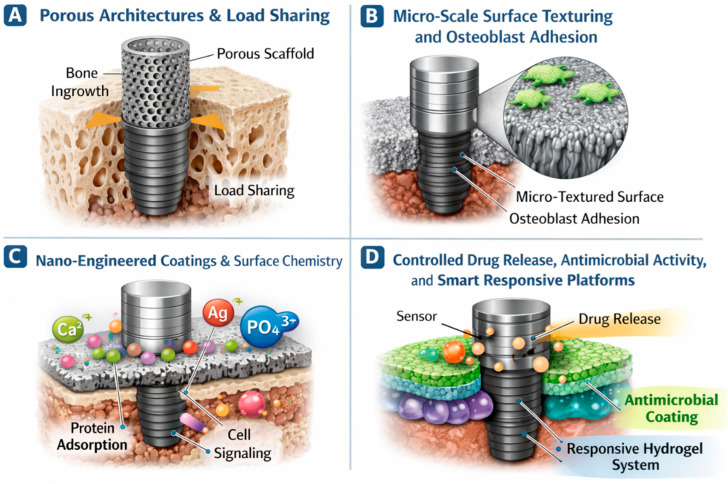
Multiscale fabrication strategies for implant interface engineering, illustrating macro-, micro-, nanoscale, and hybrid approaches for mechanical integration and biological functionality. (figure created by the authors).

Multiscale interface engineering not only relies on biological principles but also on materials platforms that enable hierarchical modification [98]. Orthopedic and dental implants should have a fair balance of mechanical stiffness, structural durability, surface reactivity, and manufacturability [99,100]. On one extreme, macro-level architectures must have bulk strength and load-sharing capability; but on the other extreme, nano-level functionality needs chemical precision and biomolecular stability [101]. The multiscale fabrication strategies underlying implant interface engineering are illustrated in Figure 4.

### 4.1. Macroscale Porous Architectures

Macroscale engineering is concerned with the way the implants interact with bone mechanically, with the redistribution of load, and by providing ways in which bone may grow [102]. Porous metals, initially in orthopedics as sintered acetabular cup finishes, have since been developed as an entirely built architectural structure, using additive technologies [103,104,105]. With these scaffolds, mechanical stiffness can be programmed to act more like trabecular bone, thereby decreasing stress shielding and increasing surface area for cellular infiltration [106]. Three-dimensional macro-porosity facilitates angiogenesis and offers channels of bone replacement as opposed to simple apposition, in that fixation relocates to the interface [107].

Another macroscale development is that of graded porosity and stiffness zoning. By gradually fading load peaks and eliminating mechanical jumps between implant and bone, gradients help to provide biologically desirable strain conditions [108]. Macro-geometry has also been shown to affect primary stability, thread engagement, and insertion torque in dental fixtures—mechanical requirements needed to achieve early osseointegration [109]. All the macro-level designs form a template structure to superimpose biological functions with micro- and nanoscale strategies.

### 4.2. Micro-Texturing and Surface Roughness

The earliest evidence-based biological intervention that became widely used was micro-scale engineering, which was based on evidence that osteoblasts are more attracted to defined roughness than polished surfaces [110,111]. Subtractive structuring, including sandblasting and acid-etching, creates repeatable topographies that retain fibrin matrices, increase osteoid deposition, and restrict shear movement at the bone–implant interface [112]. Additive processes, such as plasma spraying and micro-structured coating, have further widened the list of textures and have formed micro-recesses, allowing for bone infiltration [113].

Micro-texturing is favorable for mechanical stability as the rough surfaces increase the coefficient of friction and interfacial shear strength, which in turn decrease the chances of micromotion [114]. It is also shown that micro-features play a role in early immune occurrences, which confine inflammatory signals and stimulate tissue maturation in the future [25]. Micro-structures placed in the collar and abutment areas in dental platforms offer anchorage to connective tissue and enhance the possibility of soft-tissue sealing, which is a key point of defense against oral biofilms [115]. The microenvironment, therefore, plays the role of mediating the continuum between mechanical integration and molecular signaling [116].

### 4.3. Nano-Patterning and Surface Chemistry

The biochemical language of healing is addressed through nanoscale modification [117]. Proteins arrive before cells, so nanoscale geometry affects the conformation and activity of adhesion molecules, osteogenic factors, and immune regulators [118]. Integrin binding through nanopatterned layers of oxide, molecular coating and peptide sequences can enhance cytoskeletal tension and transcriptional activities associated with osteogenesis and angiogenesis [119]. These processes reduce reliance on the biological diversity of the host, replacing passive acceptance of wounds with artificial and engineered cell-instructive bias.

Nanoscale influence can be enhanced through chemical functionalization. Calcium, phosphate, magnesium, silver, or zinc are released on ion-releasing platforms to aid in osteogenic mineralization and antimicrobial defense [28]. Surface hydrophilicity, charge distribution, and catalytic reactivity are utilized to optimize the kinetics of protein adsorption [120]. Nano-engineered collars for dental applications promote fibroblast growth, collagen orientation perpendicularity, and a stronger epithelial boundary [121]. Micro-texturing and nano-chemistry determine the architectural scaffold and molecular tone of immune and microbial resistance [122].

### 4.4. Hybrid and Smart Multiscale Platforms

Hybrid platforms combine macro-mechanical scaffolding, micro-topography, nano-signaling, and biochemical modulation into single-structure designs that function more like adaptive tissue [123]. Multiscale porosity with localized nano-coatings is now achievable through additive manufacturing [124]. Biofunctionalized surfaces feature antimicrobial barriers, osteogenic peptides, and controlled-release reservoirs, impacting healing pathways over weeks rather than hours [125].

Responsive interfaces are a step ahead conceptually: surfaces that detect biological changes, such as pH change or inflammatory signals, or mechanical force and adjust the local release of bioactive species [126,127]. These strategies target failure mechanisms driven by physiological dysregulation: aseptic inflammation, micro-gap bacterial invasion, soft-tissue abrasion, or impaired bone remodeling [128]. By leveraging architecture, surface physics, and biochemical education, hybrid platforms create interfaces that maintain active mechanical and biological balance [129]. Table 3 provides a comparative overview of fabrication strategies across scales.

## 5. Clinical Applications in Orthopedics and Dental Implantology

**Figure 5:**
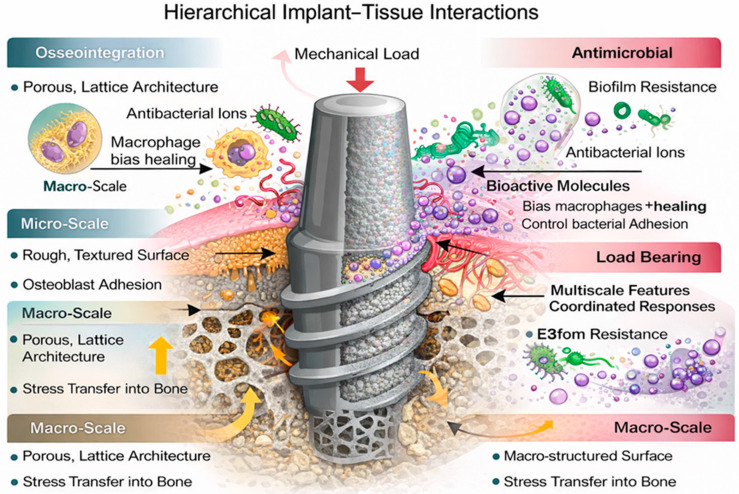
Conceptual schematic illustrating multiscale implant interface engineering, highlighting macro-porous architecture, micro-rough surfaces, and nano-bioactive chemistry that promote osseointegration, soft-tissue sealing, antimicrobial activity, and mechanical load transfer (figure created by the authors).

Multiscale interface engineering can only be translated into clinical value through its architectural and biochemical strategies to enhance stability, tissue protection, and long-term functionality [2]. The biological requirements of orthopedic and dental implants differ and overlap, including intensive anchorage of loads to deep bone, microbial defense ability of peri-implant soft tissues, anti-biofilm colonization responses, and decades of mechanical stability [74]. An engineered surface should direct bone growth, attach connective tissues, inhibit immune dysregulation, and decrease the chance of biologic or mechanical failure [134]. This section measures the applications by determining the connection between hierarchical design and clinical performance requirements. Figure 5 illustrates the concept of multiscale implant interface engineering and its biological implications.

### 5.1. Osseointegration and Bone Ingrowth

Osseointegration cannot be discussed as a single structural event but as a progressive biological process determined by retention of fibrin, recruitment of osteogenic cells, deposition of a matrix, and mineralization [135]. There is further development of the continuum under the new, constantly shifting loads [136]. The macro-porous and lattice-based structures increase primary mechanical stability by spreading the load into the trabecular spaces and also offer three-dimensional pathways to osteoblast penetration and vascular infiltration [137]. Fixation develops as bone colonizes these pores, moving to fixation by volumetric anchorage to strengthen shear resistance and diminish the potential of loosening [138].

Micro-texturing enhances the initial interface by dictating the interaction between fibrin and enhancing the structure of the osteoid matrix [139]. The surface energy is augmented, leading to rapid osteoblast settlement and growth, which facilitates earlier mineralization [106]. Nanoscale characteristics also control protein conditioning and integrin binding, creating greater intracellular tension and more secure osteogenic commitment [140]. Combined, these hierarchical traits decrease the period of mechanical weakness and decrease host biological variability at least in weakened bone.

### 5.2. Soft-Tissue Integration and Barrier Formation

Peri-implant soft tissue is not only a covering but also a biologic barrier [141]. In dentistry, the transmucosal area should be able to stop the migration of microorganisms in the peri-implant sulcus and stabilize the seal of the epithelium [142]. Micro-texturing promotes fibroblast anchorage and collagen fiber organization that disorders the peri-implant interface, which is a passive scar-like surface, into a structured connective-tissue cuff [143]. Nanoscale chemistry promotes epithelial stratification and affects immune functions that control bacterial access and wound remodeling [74]. The outcome is an augmented obstruction of the mucosa that is less prone to breakdown.

Similar problems are encountered with orthopedic applications in the use of transcutaneous or soft-tissue-exposed hardware [144]. In cases when epithelial and connective tissues fail to establish a solid attachment, the access of bacteria grows and inflammation remains [145]. Hierarchical engineering is currently being investigated to reduce epithelial recession, offer mechanical friction to tissue slip, and maintain immune balance at the interface [54]. As expected, a predictable soft-tissue seal then acts as a biological portent of mechanical fixation [146].

### 5.3. Infection-Resistant Surfaces

Microbial colonization has been one of the most disastrous effects on the survival of implants [147]. After the bacteria have attached themselves to the surface and created structured biofilms, it becomes hard to eliminate them even with systemic antibiotics, and in most cases, implant removal is necessary [148]. Hierarchical engineering destroys this progress at several levels. Macro-geometry has the potential to cut down on dead space and stagnant fluids; micro-structure can manipulate surface energy to inhibit adhesion of bacteria; and nanoscale chemistry can directly disrupt bacterial binding or metabolic functions [149]. Biological extensions of the physical design can be ion-release platforms, antimicrobial peptides, or biofilm-resistant surfaces [150].

Antimicrobial engineering has the highest potential in combination with immune modulation [151]. When the nanoscale cues support the pro-healing phenotype biases on macrophages, there is less inflammatory cycling, and the host environment becomes less permissive to chronic inflammatory infection [152]. These antimicrobial approaches have been shown to support the stability of epithelial and connective tissues in dental uses to reduce the risk of peri-implantitis [153]. In orthopedics, especially in revision cases or trauma reconstruction, the design of the interface so that it will not be contaminated initially may make the difference between successful fixation and eventual explantation [154].

### 5.4. Mechanical Stability in Load-Bearing Sites

Orthopedic implants experience continuous cyclic loading, which affects bone remodeling over the lifespan of the device [155]. Macro-engineered stiffness gradients inhibit stress shielding through facilitated transfer of physiological strain, inhibiting the region’s bone resorption [156]. Compressive forces are shared by architecture lattices, shear concentrations are diminished, and fixed stipulations are transformed to a dynamic load-sharing interface [157,158]. In dental systems, thread geometry and macroscale anchorage are not controlled, which has an influence on the insertion torque, early anchorage, and the ability to be loaded immediately [159].

Micro- and nano-features enhance mechanical interactions, supported by the enhancement of the biological shear interface [160,161]. The more surface features are surrounded by the matrix mineralizing and integrating around them, the more resistant the implant to micromotion [162]. Mechanical exposure to biological readiness is thus required in functional integration [163]. Hierarchical surfaces enhance that synchronization so that in dentistry, they can be loaded earlier, and in orthopedics, they reduce instability-induced osteolysis [164]. Finally, a functional fixation occurs, whereby bone is subjected to mechanical forces instead of being reconstructively remodeled [165]. A summary of multiscale engineering strategies and their prioritized clinical outcomes is presented in Table 4.

## 6. Translational and Clinical Integration

As multiscale interface platforms have transitioned from the laboratory development phase, their effectiveness is dependent on successful progression through preclinical trials, production reliability, and utility in surgical and prosthodontic procedures [171,172]. It is not only necessary to demonstrate biological superiority, but the engineered surfaces must also withstand mechanical instrumentation, sterilization, regulatory scrutiny, and real-time use by clinicians [173]. Therefore, it is crucial to ensure that translational integration maintains a seamless connection between biological potential, industrial viability, and procedural reliability.

### 6.1. From Laboratory Surfaces to Preclinical Implant Models

Laboratory findings provide initial evidence of osteogenic signaling, immune regulation, and antimicrobial functionality, but these results must be validated in living systems with mechanical loading, vascular access and immune variability [174]. Preclinical bone models can assesses the early stages of osseointegration under dynamic loads, while large-animal experiments can determine if macro-porous scaffolds can be vascularized and promote volumetric bone regeneration [175].

Soft-tissue formation, seal integrity, and biofilm resilience are aspects of dental preclinical models that cannot be replicated in vitro [176]. These studies also investigate the impact of insertion torque, thread geometry, and immediate loading on regenerative timelines [177]. A critical focus is on the transition to nanoscale features and biochemical coatings that remain intact post-surgical placement, drilling heat, or irrigation [178]. Many potential clinical chemistries developed in the lab are ineffective if they degrade quickly, dissipate prematurely, or fail to withstand shear forces during insertion [179].

Effective preclinical studies demonstrate three key out comes: rapid biomechanical fixation, stable soft-tissue margins, and an immune coating that prevents prolonged inflammation [180]. Only when these criteria are met can a platform be moved towards scale-up in manufacturing.

### 6.2. Manufacturing Scale-Up of Engineered Interfaces

The process of developing surface concepts for manufacturing scale-up involves transforming these concepts into production-ready products [181]. Additive manufacturing of macro-porous structures must ensure consistency in pore geometry, strut thickness, mechanical stiffness, and powder processing [182] to maintain fixation and strain transfer [183]. Micro-structured surfaces created through blasting or etching are sensitive to variation in roughness or wettability with slight changes in chemistry or exposure time [184]. Functionalization at the nanoscale, such as molecular coatings ion delivery or antimicrobial properties, must survive sterilization conditions and retain its effectiveness [185].

Validation in an industrial setting involves integrating metrology, mechanical testing, surface chemistry analysis and accelerated aging [186]. Implants introduced into clinical practice should perform similarly to preclinically tested implants that have undergone qualification processes [187]. Regulatory agencies require reports on long-term stability, elution profiles and host compatibility for bioactive and controlled release platforms [182]. Production is tailored to meet both biological and compliance standards.

### 6.3. Integration with Surgical and Prosthodontic Practice

Highly engineered surfaces must be user-friendly within the clinical workflows familiar to clinicians [162]. Orthopedic surgeons require consistent mechanics for insertion, broaching, reaming compatibility, and either press-fit or cementless fixation [162]. Macro-porous constructs must be robust enough to provide biological advantages without being too fragile to withstand impact or too soft to resist compressive forces [188]. In dentistry, thread geometry, insertion torque, collar positioning, and transmucosal height selection align mechanical stability with soft-tissue adaptation [189].

**Figure 6:**
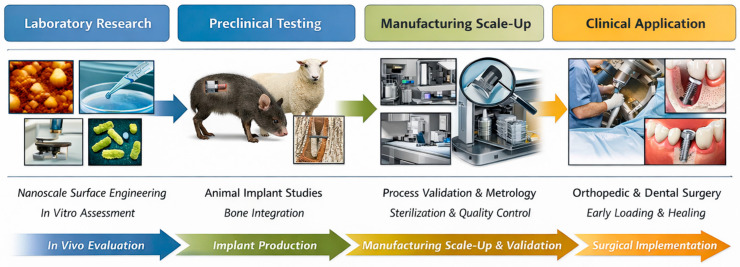
Conceptual schematic illustration for the translational pathway of multiscale engineered implant interfaces from laboratory surface design and preclinical validation to manufacturing scale-up and clinical implementation (figure created by the authors).

Operative integration also involves early implant loading [190]. By using hierarchical interfaces to bond bones, clinicians can reduce immobilization times, provided mechanical threshold are met [191]. Respectful behavior towards epithelial and connective tissues is crucial for achieving optimal emergence profiles and abutment transitions in soft-tissue areas [28]. The ultimate translational outcome is the reduction in chair-time uncertainty, improved predictability, and decreased biological risk through engineered surfaces that simplify procedures [120]. The pathway from laboratory surface design to clinical implementation is illustrated in Figure 6.

The most significant needs at every translational phase are presented in Table 5.

## 7. Key Challenges and Limitations

Even though multiscale interface engineering has reached a high level, its digital clinical efficacy is limited by biological variabilities, mechanical viability threats, antimicrobial ambiguities, and translational challenges [195]. Long-term effects with the aid of host reactions, loading conditions, microbial ecology, and regulatory tolerance rely upon the presence of macro-, micro-, and nanoscale cues that encourage early attachment [196]. These qualities indicate that the engineered hierarchy enhances the chances, rather than the certainties, of effective integration.

### 7.1. Biological Variability and Host Response

Engineered interfaces cannot entirely control the biological variability of patients receiving orthopedic surgery or dental reconstruction [197]. The process of osteogenic lineage commitment, angiogenic support, and balanced immune activation is required for osteogenic integration and is impaired in conditions such as osteoporosis, diabetes, chronic inflammation, tobacco exposure, and dysbiosis [198]. In these cases, macro-porosity may fail to trigger sufficient vascular invasion, micro-texture may fail to counteract delayed formation of the matrix, and nanoscale biochemical cues may be masked by a systemically deviated immune response [199].

Soft-tissue sealing is also highly susceptible to patient-specific biological conditions [200]. Connective tissue attachment depends on the adhesion of fibroblasts and the structure of collagen fibers, which may be disrupted by inflammatory cytokines, defective vascularity, and microbial activity [201]. Even advanced nano-surfaces may be incapable of completely inhibiting the polarization of macrophages in some systematic conditions to pro-inflammatory phenotypes [202]. Therefore, biological heterogeneity still affects the development of the process of osseointegration, the protection of soft tissues, and the long-term performance of implants.

### 7.2. Long-Term Mechanical Durability and Degradation

The structures of bone ingrowth become more porous and lose stiffness, potentially losing fatigue strength if architectural thresholds are exceeded [203]. Straining of the lattice strut repeatedly can result in deformation, local stress concentration or local instability, particularly in the stem of a femur or acetabular component [204]. Micro-rough surfaces increase interfacial friction and shear strength, which may be insufficient to overcome an overall undesirable construct, mechanically suboptimal insertion, or control of para-functional forces [205].

Nanoscale and chemical modifications present additional challenges regarding to durability [206]. The nanostructure of oxide coating or molecular coating may degrade during steam sterilization, insertion torque, or fluid exposures [207]. Ion-releasing surfaces may deplete too quickly, reducing biological activity in the long term [208]. Mechanical polishing or stripping of hierarchical features in dentistry may be achieved through platform switching, abutment modification, polishing, and prosthetic adjustments [209]. Therefore, work hardening, manipulation, and cyclic fatigue pose a threat to hierarchical surface stability even with successful early fixation.

### 7.3. Infection Control and Biofilm-Resistance Limitations

Although nanochemical approaches can decrease bacterial adhesion, they may struggle to prevent the formation of biofilms in the presence of saliva or hematogenous contamination [210]. Surface modifications may slow early colonization, but established multispecies communities can overpower passive antimicrobial protection [211]. The use of ion-release platforms carries a risk of concentration-dependent host-cell toxicity, and the antimicrobial effectiveness of ion-release platforms may decrease as ions dilute in vivo [212].

**Figure 7:**
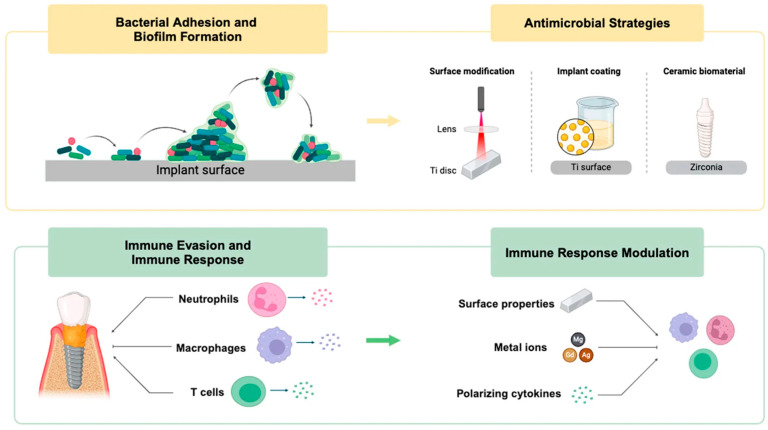
Biofilm establishment on implant surfaces and interaction of the immune system, inflammatory reactions, and antimicrobial and immune-modulating surface-based approaches. Adapted from Ref. [216]. CC BY 4.0 © 2024 MDPI, Basel, Switzerland.

The instability of soft tissues further increases the risk of infection. In cases where epithelial and connective tissue barriers are compromised, microbial invasion raises the risk of peri-implant inflammation, leading to bone loss in dentistry and chronic infection in orthopedics [213]. In such scenarios, systemic antibiotic treatment may not be entirely effective, as biofilm communities are metabolically supportive [214]. Safety risks arise from both the risks of biological toxicity and the challenges of long-term antimicrobial control [215]. Figure 7 illustrates the established mechanism of biofilm development and immune response at the implant surface.

### 7.4. Regulatory and Clinical Adoption Barriers

The translation of hierarchical interface technology requires not only biological success but also the ability to meet manufacturing consistency, regulatory scrutiny, and clinical usability [217]. The regulators require durability information, coating adherence, and long-term safety data on controlled-release or biochemical agents [218]. Such demands hinder the market penetration of implants coating antimicrobial ions, peptides, or responsive chemistry [219]. Additionally, cost-based evaluations hinder adoption as health care systems might be unwilling to reimburse surface improvements until a reduction in revision rates is proven [220].

Workflow compatibility is also needed by clinicians. The surgeons require predictable insertion mechanisms, resistance against revision forces, and compatibility with current instrumentation [221,222]. Prosthodontists expect collar areas to maintain torque and polish and manipulate prosthetics without affecting nanoscale activity [223]. If engineered surfaces introduce additional learning curves, sterilization limitations, or cost increases, adoption may be slower, even with favorable biological benefits [224]. The complexity of regulations, financial risks, and procedural conservatism work together to limit widespread implementation [225]. A comparative summary of these key biological, mechanical, microbial, and regulatory limitations is presented in Table 6.

## 8. Comparative Clinical Outcomes and Evidence Synthesis

The field of clinical translation is where multiscale interface engineering is put to the test: Do hierarchical surfaces provide better survival, tissue stability, and complication rates in actual patients? The existing evidence is not unanimous: early fixation seems to be better, but long-term randomized evidence is scant, and the results are usually confounded by patient risk factors [229]. The following subsections provide a summary of what is known, how engineered implants would perform in comparison to conventional surfaces, and what meta-analyses have to say about long-term performance.

### 8.1. Clinical Performance of Multiscale-Engineered Orthopedic Implants

The promising results of osseointegration in early- and mid-term clinical series in additively manufactured highly porous titanium acetabular and femoral components are associated with encouraging radiographic features and satisfactory early survivorship in primary and revision arthroplasty cases [230]. Systematic reviews of additive-manufactured porous implants conclude that the volumetric bone ingrowth and initial stability of problematic reconstructions can be enhanced by customization of pore size, porosity, and lattice structure [231].

The bioactive coating that has traditionally been used is plasma-sprayed hydroxyapatite (HA), which has a long history of clinical use in the increased rates of early bone apposition and fixation in a small percentage of regions [232]. Nevertheless, the durability of the coating, delamination, and unreliable acetabular performance (in comparison to the femur) dampen the excitement about its universal application [233]. This contradictory long-term evidence points to the fact that premature radiographic osseointegration does not always lead to decades of mechanical stability unless the characteristics of coating and implant mechanics are tightly controlled [234].

Low early migration and radiographic bone contact are observed in most cohorts of clinical case series of fully porous 3D-printed cups and stems [169]. The revision rates and infection rates in complex revision populations are not negligible, which indicates that porous design only helps solve significant fixation issues, yet still requires precise surgical training, infection and disease prevention, and extended follow-ups [235].

### 8.2. Evidence from Dental Implantology and Peri-Implant Tissue Response

Moderately roughened and micro-textured dental implant surfaces, according to clinical evidence, are better in bone contact, quicker to stabilize, and have slightly less marginal bone loss than machined surfaces [236]. These merits allow for shorter loading practices in a great number of patients [237]. Several manufacturer-level meta-analyses and independent systematic reviews have further supported the clinical advantages of regulated micro-roughness to foreseeable osseointegration [238].

**Figure 8:**
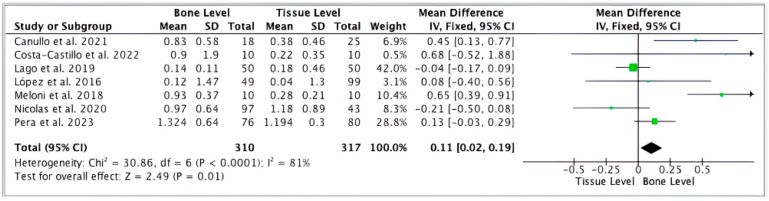
Clinical meta-analysis forest plot comparing marginal bone loss between bone-level and tissue-level dental implants. Reprinted from Ref. [242]. CC BY 4.0 © 2024 MDPI, Basel, Switzerland.

The relationship between the roughness of the surface and peri-implant disease is complicated [239]. Although rough surfaces might allow for increased bone contact, they can also enhance the available area of contact for bacteria should there be no good sealing of the soft-tissues, and most importantly, the long-term incidence of peri-implantitis depends critically on patient factors (periodontitis history, oral care, smoking) and the management of the prosthetics and not on the overall surface topography [240]. Recent systematic syntheses have underlined the fact that a patient’s risk profile is a foremost predictor of peri-implantitis and implant failure despite advanced surfaces in application [241]. A comparative clinical evaluation of marginal bone loss between bone-level and tissue-level implants is represented in Figure 8.

### 8.3. Systematic Comparisons Versus Conventional Implant Surfaces

When compared directly to either multiscale or hybrid surfaces or conventional machined or simply blasted implants, early mechanical and biological results are often more favorable [243]. Nevertheless, most of such studies are limited by short follow-ups, heterogeneous cohorts, differences, and low methodological rigor [236]. Subsequently, when disparate trials are aggregated using meta-analyses, the effect size is usually quite modest with large confidence intervals, especially when using a long-term endpoint, like peri-implantitis or aseptic loosening [244]. In orthopedics, even though the use of HA coatings and porous structures often results in better initial fixation, there is no consistent evidence of long-term survivorship, and it is still contingent on the design of implants and the surgical setting [29,245].

### 8.4. Meta-Analytic Signals, Success Rates, and Complication Patterns

In both meta-analyses and registry reports, the overall survival of implants with modern systems is high, irrespective of the class of surface, although there are some common trends [195]. Primarily, engineered multiscale surfaces predictably increase early fixation parameters and radiographic bone contact [246]. Second, long-term survival advantages are based on the compatibility of the surface technology, mechanical design, coating stability, and clinical administration [196]. Third, patient-level variables such as periodontitis history, smoking, and systemic disease can oftentimes have a greater effect on the complications than the very type of the surface [247]. Collectively, the existing evidence is in favor of multiscale engineering as a concept of minimizing early vulnerability windows and enhancing initial outcomes [248], against the backdrop that, to implement a significant decrease in long-term failure, effective manufacturing, standardized surgical guidelines, and prolonged follow-up are essential [249]. A summary of clinical evidence comparing multiscale, hybrid, and conventional implants across different domains is presented in Table 7.

## 9. Industrial and Translational Perspectives

Commercial success is the result of multiscale interface engineering and its ability to enhance patient outcomes at the population level [253]. Technical maturity (reliable manufacturing, sterilization durability), economic feasibility (lower revision cost, reduced treatment time), ease of regulation, and stakeholder cooperation that minimizes the risk of development influence industry adoption [254].

### 9.1. Industrial Adoption of Advanced Surface-Engineered Implants

The adoption of hierarchical surfaces by the industry is progressing most rapidly in areas where manufacturing platforms, particularly metal additive manufacturing and controlled surface finishing, are present [255]. It depends on the reproducibility of macro-porous structure and the reliability of micro-/nano-topography [256]. Additive manufacturing (e.g., selective laser melting) enables complex lattice structures and patient-specific geometries, becoming popular in acetabular cups, metaphyseal cones, and bespoke reconstructions [257]. Adoption is uneven, with high-volume products with standard workflows more readily incorporating multiscale features than highly regulated or low-volume applications, which are more expensive to qualify and validate [258].

Scale-up involves rigorous process control where powder chemistry, laser settings, post-processing, surface finishing, and inspection are confirmed to ensure that the desired hierarchical structure of macro-porous geometry, microroughness, and nanoscale chemistry is preserved through batches [259]. Firms with established additive-manufacturing production lines and in-house metrology have seen the most clinical adoption, although persistent variability remains a hindrance to regulatory assurance and slow clinical adoption [260].

### 9.2. Cost–Benefit and Economic Considerations

Engineered surfaces can minimize costly complications, particularly revisions [261]. In orthopedics, a revision procedure, especially a two-stage septic revision, is significantly more expensive than primary implantation, making even minor savings worthwhile [262]. In dentistry, fewer peri-implant complications reduce retreatment and prolong the life of the prosthesis [263]. Economic models compared the increased cost of surface engineering with savings on prevented revisions, reduced rehabilitation, and clinic visits [264]. Registries and health-economic analyses show that revisions remain the biggest contributor to long-term costs, supporting the cost-efficiency of durable interface upgrades in high-risk or complex cases [265].

The business case is context-sensitive; the clinical benefit of engineered surfaces may be smaller in low-risk primary cases [266] but greater in revisions, oncologic reconstruction, or severely osteoporotic bone [267]. This leads payers and hospitals to demand real-world evidence or registry information before consenting to the high prices of these technologies [268].

### 9.3. Regulatory Frameworks and Approval Pathways

Regulation is a significant bottleneck for surfaces engineered with bioactive chemistry, controlled-release agents, or living components [269]. Devices are grouped by their mode of action, usually mechanical [172], with active surfaces considered medical devices and surfaces with pharmacologic or biologic action classified as combination products requiring additional evidence [171]. Regulatory guidance is based on production quality, leachables, sterility, and long-term stability of coatings or ion release systems [270]. In the U.S., FDA combination-product and CGMP frameworks govern technologies bordering on devices, drugs, and biologics [271].

The Medical Device Regulation (MDR) in Europe has raised demands on clinical evidence and post-market surveillance, with recent amendments easing some processes [171]. Manufacturers must strategize regulatory paths early, defining the main mode of action, developing durability and biocompatibility evidence, and establishing post-market surveillance for long-term performance tracking [272].

### 9.4. Collaborative Innovation Across Dentistry and Orthopedics

**Figure 9:**
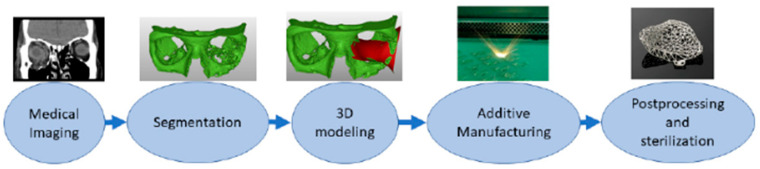
An additive manufacturing system demonstrating the process of digital design through fabrication and post-processing of medical and dental implants. Reprinted from Ref. [256]. CC BY 4.0 © 2021 MDPI, Basel, Switzerland.

Effective translation of multiscale interface technologies requires collaboration due to technical, clinical, and regulatory complexities [273]. Public–private collaborations, consortia, and common registries facilitate standardized testing and multicenter clinical trials, creating larger datasets persuasive to regulators and payers [274]. Non-competitive standards and shared results, through open-innovation models, reduce duplication and enhance quality [275]. Governments and industry organizations are forming translational hubs to co-create manufacturing platforms, validation pipelines, and clinical-trial systems for adopting emerging surface technologies [276]. These collaborative models are applicable to common issues in dentistry and orthopedics, e.g., antimicrobial surfaces or fatigue testing of porous lattices [277]. Similar approaches and comparative registries expand the evidence base and distribute the cost of long-term follow-up. A workflow example of additive manufacturing for orthopedic and dental implants is presented in Figure 9.

The industrial needs, translational obstacles, and common mitigation measures related to multiscale interface technologies are summarized in Table 8.

## 10. Future Roadmap (2025–2035)

Future areas of multiscale interface engineering will focus on developing surface architecture fidelity, balancing biomechanical interactions, resistance to biofilm and generating long-term clinical data [283]. It will be necessary to tightly integrate manufacturing science and translational biology to achieve these goals, and regulatory predictability should be obtained using real-world clinical analytics instead of laboratory proxies.

### 10.1. Short-Term Goals (2025–2027)

Short-term progress will be based on reproducible surface production and quantifiable biological results [284]. Additive manufacturing platforms are expected to transition to standardized lines of implants with proven pore geometry, stiffness gradients, and fatigue resistance [285]. Dental and orthopedic suppliers will also need to streamline micro-roughness protocols to reduce batch variability, stabilize surface wettability, and ensure that nanoscale features resist machining, sterilization, and insertion torque [286].

Short-term biological priorities include enhancing early immune compatibility by inhibiting pro-inflammatory macrophage activation and promoting an anti-inflammatory, pro-healing macrophage phenotype [287]. New collar designs resistant to infections can be integrated into dental practice to prevent microbial penetration of peri-implants without systemic antibiotics [288]. Additionally, regulatory and payer frameworks are likely to require increased post-market surveillance, i.e., manufacturers will need to coordinate product release with outcome tracking based on registries [289].

### 10.2. Mid-Term Objectives (2028–2031)

Mid-term goals extend beyond interface stability to adaptive biological control [290]. Controlled-release chemistries are expected to be engineered onto surfaces to remain effective during the early wound stabilization phase of tissue remodeling [291]. In orthopedics, more porous and gradient lattice scaffolds may be designed to accommodate full-thickness vascular networks, allowing for bone growth in large defects or complex revision cases.

Smart dental collars could potentially help regulate the interface between tissue and implants, reducing soft-tissue loss due to dysbiosis [292]. Improved regulations categorizing combination products are likely to enable manufacturers to develop evidence earlier in the development process [271]. With the growth of shared registries, benchmarking between centers will become more important for identifying clinically significant variations in revision rates and peri-implant complications.

### 10.3. Long-Term Vision (2032–2035)

In the long term, advances in multiscale interface engineering should result in implants that resemble regulated biological environment, incorporating hierarchical structure, biochemical signaling, and intelligent feedback [284]. Mechanically adaptive stems and cups in orthopedics could potentially adjust stiffness over time, preserving strain transfer during bone remodeling and reducing stress-shielding-induced resorption [293]. Fully integrated lattice vascular systems could allow for biological regeneration of large segmental defects without the need for graft augmentation [294].

**Figure 10:**
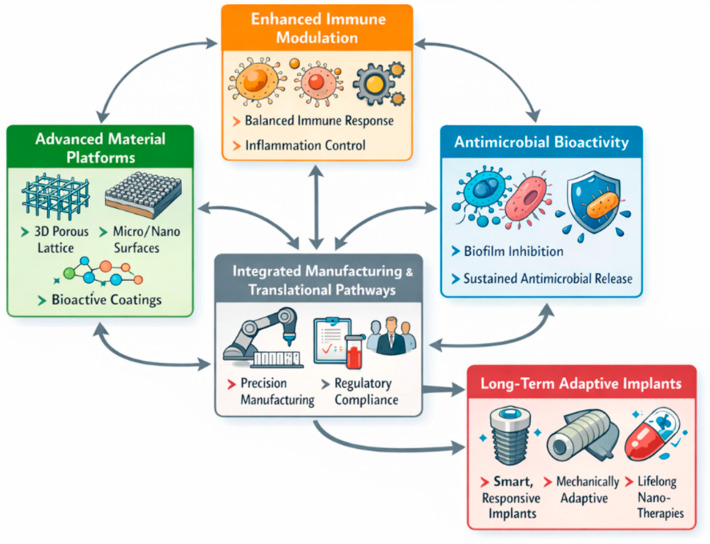
Conceptual roadmap for next-generation multiscale implant interfaces, highlighting advanced materials, immune modulation, antimicrobial bioactivity, and translational manufacturing pathways (figure created by the authors).

Dental systems may evolve into immune-interactive transmucosal systems that maintain the sealing of epithelia and counteract microbial risks automatically [295]. Engineered nanoscale reservoirs could provide antimicrobial properties for years instead of weeks. Clinical confidence at this stage will be based on multi-national registry analytics, stable surface chemistries, and verified cost–benefit indicators showing fewer clinical revisions and complications [296]. Implants would no longer be passive devices but dynamically orchestrated micro-environments that sustain tissue integrity throughout their functional lifespan [297]. The strategic pillars and future directions of multiscale implant interface engineering are illustrated in Figure 10.

## 11. Conclusions

Multiscale interface engineering has transformed the design of implants from mechanical fittings to biologically active, hierarchically structured systems. Recent findings in laboratory, translational, and early clinical research suggest that sustainable success requires a combination of structural, mechanical, and biochemical signals across scales of various lengths. What was initially aimed to enhance osseointegration rates has now evolved to focus on long-term tissue stability.

### 11.1. Summary of Key Advances

Recent advancements can be summarized in four main accomplishments. Firstly, volumetric bone development and load distribution are no longer surface-based, thanks to the use of macro-porous and architected scaffolds. Secondly, early biological vulnerability has been reduced through micro-textured topographies and regulated roughness promoting fibrin anchorage, osteoblast adhesion, and initial extracellular matrix formation. Thirdly, nanoscale patterning and biochemical functionalization allow for control over protein adsorption, integrin signaling, immune polarization, and epithelial behavior—functions previously unattainable with machined surfaces. Lastly hybrid platforms with antimicrobial chemistry, controlled-release agents, and adaptive mechanics signify a shift towards biologically instructive systems rather than passive implants.

### 11.2. Remaining Scientific and Clinical Gaps

Despite these advancements, there are still four gaps to address. Biological variability poses challenges in predicting responses, especially in patients with conditions like osteoporosis, diabetes, immune-dysregulation, or microbiome imbalances. Long-term sustainability remains uncertain, as porous scaffolds and nanoscale coatings must withstand decades of loading and clinical use. Microbial control is still a challenge, as biofilms can persist despite surface-energy manipulations or temporary ion-release techniques. Additionally, clinical evidence is primarily short- to mid-term, lacking long-term follow-up data. These gaps suggest that hierarchical surfaces may lead to increased chances, though not guarantees, of long-term biological and mechanical success.

### 11.3. Recommendations for Future Research

Future studies should utilize standardized models to assess immune polarization, angiogenic maturation, soft-tissue adhesion, and osteogenic outcomes. Orthopedic research should focus on improving lattice fatigue behavior, optimizing pore structure for better vascularization, and testing biologic agents in high-risk revision surgeries. Dental research should explore epithelial-sealing techniques, controlled-release antimicrobial collars, and long-term peri-implant bone mapping.

In conclusion, advancements in hierarchical collars and biologically active surfaces in dentistry can improve mucosal barrier preservation, reduce peri-implant inflammation, and allow for earlier and more predictable loading. The surface architecture, bioactive chemistry, and responsive feedback holds promise for the development of self-repairing implant interfaces that can sense inflammatory disturbances, repair surface functionality, and maintain antimicrobial defenses independently. These advancements offer practical guidance for designing next-generation orthopedic and dental implants with enhanced biological integration and long-term performance.

## Figures and Tables

**Table 1:** Hierarchical features of native tissues and their functional contributions to implant-interface design.

Biological Scale	Dominant Structural Features	Functional Importance for Interface Design	Representative Engineering Translation	References
Macro level (bone and soft tissue)	Cortical trabecular transitions; anisotropic porosity; vascular channels.	Scatter loads, allows for remodeling, allows for vascular access.	Porous metals; lattice constructs; graded-stiffness architectures.	[67]
Micro level (matrix organization)	Lamellae; osteons; orientation of collagen fiber; attachment of the epithelial/lamina.	Enhances shear resistance, guides the matrix deposition, stabilizes the adhesion of soft-tissues.	Controlled surface roughness; blasted/etched textures; micro-patterned collars.	[68]
Nano level (molecular assembly)	Mineral platelets fibrils of collagen; integrin-binding ligands; collagen-protein interactions.	Modulates the protein orientation, cell differentiation, immune phenotype.	Nano-patterning; chemical functionalization; ion-modifying coatings.	[35]

**Table 2:** Evolution of implant interface technologies: from mechanical fit to multiscale bioactivity.

Development Era	Dominant Surface Strategy	Biological Outcome Achieved	Limitations Driving Next Stage	Reference
Early smooth metallic surfaces	Machined finish, low roughness, cement or press-fit fixation.	Passive tolerance, limited bone contact, slow stabilization.	Micromotion, fibrous tissue formation, high loosening rates.	[2]
Micro-rough and bioactive coatings	Sandblasting, etching, porous layers, calcium-phosphate chemistry.	Improved osteoblast adhesion, faster osseointegration, earlier loading.	Limited immune modulation, variable coating durability.	[68]
Nano-engineered and biomimetic platforms	Nano-patterning, ion release, biochemical motifs, soft-tissue bioactivity.	Integrin-mediated signaling, immune regulation, better hard/soft-tissue attachment.	Integration of multiscale hierarchy still evolving.	[97]

**Table 3:** Multiscale contribution of fabrication strategies to implant interface performance.

Scale of Intervention	Primary Fabrication Methods	Biological or Mechanical Benefit	Typical Limitations Addressed	Reference
Macro (mechanical)	Additive lattices, porous metals, stiffness gradients, thread geometries.	Improves load transfer, enhances volumetric bone ingrowth, increases primary stability.	Stress shielding, micromotion, limited contact area.	[130]
Micro (topographic)	Sandblasting, acid-etching, plasma spray, additive micro-features.	Enhances osteoblast adhesion, reinforces shear resistance, supports connective-tissue anchorage.	Slow bone apposition, limited frictional engagement.	[131]
Nano (chemical/biological)	Nano-patterned oxides, peptide motifs, ion release coatings, hydrophilic conditioning.	Modulates protein adsorption, directs cell signaling, influences immune phenotype, improves antibacterial behavior.	Inflammatory deviation, soft-tissue instability, biofilm formation.	[132]
Hybrid (smart bioactive)	Combined porosity + micro-texture + nano-functionalization, responsive coatings.	Synergistic hard- and soft-tissue integration, immune control, antimicrobial protection.	Multifactorial failure risks, biologic unpredictability.	[133]

**Table 4:** Clinical performance priorities supported by multiscale interface strategies.

Performance Domain	Key Clinical Requirement	Multiscale Contribution	Primary Failure Mode Addressed	Reference
Osseointegration and bone anchorage	Rapid, stable mineralized attachment	Macro-porosity for ingrowth; micro-texture for matrix retention; nano-cues for osteogenic signaling	Micromotion, aseptic loosening	[166]
Soft-tissue sealing	Connective-tissue anchorage and epithelial stability	Micro-structuring for fibroblast attachment; nano-chemistry for immune balance	Peri-implant boundary breakdown	[141]
Antimicrobial resistance	Suppression of adhesion and biofilm formation	Nano-chemistry, ion release, surface-energy control	Infection-driven failure	[167,168]
Mechanical endurance	Load distribution and strain transfer	Macro-stiffness gradients, lattice mechanics, frictional enhancement	Stress shielding, long-term bone loss	[169,170]

**Table 5:** Key translational requirements for multiscale interface platforms.

Translational Stage	Core Requirement	Biological or Clinical Priority Addressed	Limitation Overcome	Reference
Preclinical validation	Demonstrate bone ingrowth, immune stability, soft-tissue sealing	Aligns early healing with long-term function	Biological unpredictability in vivo	[192]
Manufacturing scale-up	Reproducible roughness, chemistry, porosity, coating durability	Ensures biological signaling remains consistent	Batch variability, coating loss	[193]
Surgical/prosthodontic integration	Predictable insertion, load readiness, tissue compatibility	Enables clinical adoption and reduced failure rates	Workflow disruption, mechanical mismatch	[194]

**Table 6:** Key limitation factors on biological, mechanical, microbial, and regulatory areas.

Limitation Category	Core Challenge	Functional Impact on Implant Interfaces	Resulting Risk	Reference
Host biology	Variable osteogenesis, impaired angiogenesis, immune deviation	Reduced bone ingrowth and soft-tissue stability	Delayed healing, peri-implant breakdown	[216]
Mechanical durability	Fatigue stress, strut deformation, coating erosion	Loss of structural stability and interfacial strength	Aseptic loosening, implant failure	[226]
Infection control	Incomplete biofilm suppression, antimicrobial depletion, toxicity	Persistent bacterial colonization and inflammation	Peri-implantitis, chronic infection	[227]
Regulatory adoption	Manufacturing, safety, economic and workflow barriers	Slow clinical integration despite biological promise	Limited usage, delayed translation	[228]

**Table 7:** Comparative clinical evidence, multiscale/hybrid vs. conventional implant surfaces.

Clinical Domain	Typical Multiscale/Hybrid Intervention	Common Clinical Evidence (Outcomes)	Key Limitations of Evidence	Reference
Orthopedics primary/revision arthroplasty	3D-printed porous titanium lattices; HA or bioactive coatings	Early radiographic osseointegration, improved primary stability; some series show low early migration	Mostly short-to-midterm data; heterogeneous designs and patient complexity	[250]
Orthopedics HA/bioactive coatings	Plasma-sprayed HA; calcium phosphate layers	Faster early bone apposition in many reports; femoral stems often perform well long term	Variability in coating durability; acetabular coatings historically mixed	[251]
Dental micro-rough implants (SLA, TiUnite, anodized)	Sandblasted/acid-etched or anodized surfaces	Higher bone-to-implant contact, earlier secondary stability, modestly lower marginal bone loss	Heterogeneous trial designs; follow-up often <10 years	[236]
Dental nano-modified/antimicrobial collars	Nano-topography; ion-release coatings; biofunctional transmucosal collars	Improved soft-tissue cell responses in vitro; early clinical promise for soft-tissue seal	Limited long-term clinical RCTs; biofilm and host factors confound outcomes	[252]
General (meta-analytic view)	Any hierarchical/topographical enhancement	Consistent early benefits; long-term survival still high across surfaces	Patient risk, surgical technique, and prosthetic management are dominant confounders	[81]

**Table 8:** Industrial and translational considerations for multiscale interface technologies.

Topic	Industry Requirement	Translational Barrier to Solve	Typical Mitigation Strategy	Reference
Manufacturing reproducibility	Tight control of AM, finishing, coating consistency	Batch variability; surface heterogeneity	Process qualification, in-line metrology, standardized SOPs	[256]
Clinical evidence and economics	Demonstrate fewer revisions/improved function	Need for long-term RCTs or registry data; payer skepticism	Pragmatic registries, targeted RCTs in high-risk cohorts, health-economic modeling	[278]
Regulatory compliance	Clear product classification; sterility and leachables data	Combination-product ambiguity; CGMP expectations	Early regulatory engagement; defined principal mode of action; stability/PK data	[279]
Sterilization and packaging	Preserve micro-/nano-features through sterilization and handling	Coating loss, nano-damage during sterilization	Validate sterilization methods, package to protect surfaces	[280]
Market adoption and clinical workflow	Surgeon familiarity, instrument compatibility	Resistance to new workflows or learning curves	Training programs, instrumentation compatibility, pilot adoption sites	[281]
Collaborative models	Shared standards and data	High cost of long-term studies and standardization	Public–private consortia, shared testbeds, open registries	[282]

## Data Availability

No new data were created or analyzed in this study. Data sharing is not applicable to this article.
